# Vedolizumab trough level monitoring in inflammatory bowel disease: a state-of-the-art overview

**DOI:** 10.1186/s12916-019-1323-8

**Published:** 2019-05-08

**Authors:** Lieven Pouillon, Séverine Vermeire, Peter Bossuyt

**Affiliations:** 1 Imelda GI Clinical Research Centre, Imeldaziekenhuis Bonheiden, Bonheiden, Belgium; 2 Department of Gastroenterology and Hepatology, University Hospitals Leuven, KU Leuven, Leuven, Belgium

**Keywords:** Crohn’s disease, Inflammatory bowel disease, Therapeutic drug monitoring, Vedolizumab, Ulcerative colitis

## Abstract

**Background:**

Therapeutic drug monitoring involves therapeutic modifications based on the measurement of drug levels and antidrug antibodies. The viability of therapeutic drug monitoring in vedolizumab-treated patients with inflammatory bowel disease remains questioned.

**Main body:**

Accumulating evidence from clinical trials and real-world data suggests that an exposure–efficacy relationship may exist for vedolizumab in inflammatory bowel disease, but results are not as straightforward as they are for anti-tumour necrosis factor-α therapy. Robust target vedolizumab trough levels are currently missing, since available data are heterogenous and prospective, interventional pharmacokinetic–pharmacodynamic studies are lacking. The positioning of vedolizumab drug monitoring in therapeutic algorithms is yet to be defined.

**Conclusion:**

Therapeutic drug monitoring has the potential to improve the outcome parameters of vedolizumab-treated patients with inflammatory bowel disease. Before the therapeutic drug monitoring of vedolizumab can be implemented in a widespread fashion, prospective studies are needed to evaluate the effect of vedolizumab dose optimisation. These studies should focus on objective disease markers and vedolizumab drug levels, and define thresholds for optimal drug exposure.

## Background

Monoclonal antibodies directed against tumour necrosis factor-α (anti-TNF) have conventionally been the cornerstone of the treatment of moderate-to-severe inflammatory bowel disease (IBD) [1]. Nevertheless, approximately one-third of biological-naïve patients experience a primary non-response to anti-TNF therapy, and secondary loss of response is seen in almost half of the patients over time [2, 3]. In the search to enlarge the therapeutic armamentarium, vedolizumab has been shown to be effective in both Crohn’s disease (CD) and ulcerative colitis (UC) [4, 5]. Although the mechanism of action of vedolizumab is not fully understood, it is presumed to act primarily by binding to α4β7 integrin, which is predominantly expressed by a subset of gastrointestinal-homing T-lymphocytes. Vedolizumab thereby prevents the interaction of α4β7 integrin with mucosal addressin cell adhesion molecule-1 (MAdCAM-1) on the surface of mucosal endothelial cells, and inhibits the migration of T-lymphocytes into the bowel tissue [6].

Therapeutic drug monitoring involves measuring drug levels and antidrug antibodies with adjustment of the dose when needed, and is particularly of interest because drug exposure – rather than the administered dose – is related to response to biologicals. Since anti-TNF therapy uses weight-based dosing, theoretically more flexibility is possible compared with vedolizumab therapy, in which a fixed dose is administered. In anti-TNF-treated patients, therapeutic drug monitoring has been shown to be a valuable tool to guide decision-making in patients with insufficient response to therapy [7, 8]. Although registered trials in CD and UC have suggested an exposure–efficacy relationship with vedolizumab [4, 5, 9, 10], the viability of therapeutic drug monitoring for vedolizumab remains less clear. To improve insights, several researchers have recently studied vedolizumab trough levels and clinical, endoscopic and histological outcome parameters in IBD patients [11–17]**.**

The aims of this mini-review are to give an overview of the currently available knowledge and future perspectives of therapeutic drug monitoring in vedolizumab-treated patients.

## Search strategy

We searched for relevant manuscripts in PubMed/MEDLINE, EMBASE (Excerpta Medica Database) and Cochrane CENTRAL, from their inception until March 5th, 2019. The following keywords were included, alone or in combination: “Crohn’s disease”, “ulcerative colitis”, “inflammatory bowel disease”, “vedolizumab”, “trough levels”, “serum levels”, and “therapeutic drug monitoring”. Relevant articles published in English were critically reviewed. Bibliographies of included articles were searched, and experts in IBD were consulted to identify additional studies. Only full papers were considered for review; data exclusively available in abstract format were not taken into consideration.

## Exposure–efficacy relationship

An exposure–efficacy relationship for vedolizumab has been suggested in clinical trials and most real-world cohorts (Table 1).

**Table 1:** Exposure–efficacy relationship of vedolizumab in inflammatory bowel disease

Reference	Design	Number of patients	Main outcome parameter	Key findings			Other findings
Clinical trials							
Rosario et al. (2017) [9]	Clinical trial (post-hoc analysis)	681 (UC); 850 (CD)	Vedolizumab TL and clinical remission status^a^ at week 6	Median (IQR) TL at week 6: clinical remission (UC)	34.7 (31.7–36.6)	*P*-value: not stated	TL at week 6 < 17 (UC) and < 16 (CD): clinical remission rates similar to placebo
				Median (IQR) TL at week 6: no clinical remission (UC)	23.7 (22.0–24.8)		
				Median (IQR) TL at week 6: clinical remission (CD)	26.8 (24.9–30.1)	*P*-value: not stated	
				Median (IQR) TL at week 6: no clinical remission (CD)	23.5 (22.4–24.8)		
Osterman et al. (2019) [10]	Clinical trial (post-hoc analysis); propensity-score-based case-matching analysis	693 (UC)	Target vedolizumab TL for clinical response/remission^b^, adjusted for confounding factors on drug clearance	Target TL at different timepoints for clinical response/remission			Week 6 was the earliest time point at which TL was consistently associated with clinical response and remission at week 14 and week 52
				Week 6	> 37.1		
				Week 14	> 18.4		
				Maintenance	> 12.7		
Real-world cohorts							
Williet et al. (2017) [11]	Prospective, observational; multicentric	16 (UC); 31 (CD)	Association between vedolizumab TL at week 0–6 and need for additional dosing in first 6 months	Cut-off TL associated with the need for additional dosing in first 6 months			
				Week 2	< 24.5	AUROC 0.62	
				Week 6	< 18.5	AUROC 0.72	
Al-Bawardy et al. (2018) [12]	Retrospective, cross-sectional; single-centre	53 (UC); 106 (CD); 12 (IBDU)	Vedolizumab TL and mucosal healing^c^	Median (IQR) TL and mucosal healing	13.7 (10–32.9)	*P* = 0.64	
				Median (IQR) TL and no mucosal healing	16.1 (7.7–27.6)		
Dreesen et al. (2018) [13]	Retrospective, observational; single-centre	66 (UC); 113 (CD)	Vedolizumab TL and clinical/biological/endoscopic effectiveness endpoints^d^ at week 14 (UC) and week 22 (CD**)**	Cut-off TL associated with effectiveness at week 14/22 (UC/CD)			
				Week 2	> 30	*P* < 0.05	
				Week 6	> 24	*P* < 0.05	
				Week 14	> 14	*P* < 0.05	
Yacoub et al. (2018) [14]	Prospective, observational; multicentric	43 (UC); 39 (CD)	Vedolizumab TL during induction (weeks 2, 6, 14) and mucosal healing^e^ within 1 year	Median (IQR) TL and mucosal healing within 1 year			TL at week 6 > 18 was the only independent variable associated with mucosal healing within 1 year (AUROC: 0.735)
				Week 2	27 (23.6–33.8)	*P* = 0.845	
				Week 6	26.8 (21.4–40.4)	*P* = 0.035	
				Week 14	16 (8–21)	*P* = 0.241	
				Median (IQR) TL and no mucosal healing within 1 year			
				Week 2	27.8 (18.1–34.5)	*P* = 0.845	
				Week 6	15.1 (13.4–23.5)	*P* = 0.035	
				Week 14	6.3 (4.5–15)	*P* = 0.241	
Ungaro et al. (2019) [15]	Prospective, cross-sectional; multicentric	116 (UC); 142 (CD)	Vedolizumab TL and corticosteroid-free clinical and biochemical remission during maintenance therapy^f^	Median (IQR) TL and steroid-free clinical and biochemical remission	12.7 (8.4–19.4)	*P* = 0.002	Patients with TL > 11.5 were 2.4 times more likely to be in corticosteroid-free clinical and biochemical remission
				Median (IQR) TL and no steroid-free clinical and biochemical remission	10.1 (5.9–15.2)		
Pouillon et al. (2019) [16]	Retrospective, cross-sectional; single-centre	31^g^ (UC)	Vedolizumab TL and histological healing^h^	Median (IQR) TL and histological healing	31.5 (25–49.1)	*P* = 0.02	TL > 25 was most optimal to predict histological healing (AUROC: 0.62)
				Median (IQR) TL and no histological healing	15 (9–26.6)		
Yarur et al. (2019) [17]	Prospective, observational; single-centre	30 (UC); 25 (CD)	Vedolizumab TL during induction (weeks 2, 6, 14) and steroid-free endoscopic remission at week 52^i^	Median (IQR) TL and steroid-free endoscopic remission at week 52			
				Week 2	24.8 (23–28)	*P* = 0.005	
				Week 6	25 (17–28)	*P* = 0.016	
				Week 14	11 (7–17)	*P* = 0.42	
				Median (IQR) TL and no steroid-free endoscopic remission at week 52			
				Week 2	20 (18–25)	*P* = 0.005	
				Week 6	17.3 (10–24)	*P* = 0.016	
				Week 14	8 (6–14)	*P* = 0.42	

*AUROC* area under the receiver operating curve, *CD* Crohn’s disease, *IBDu* indeterminate inflammatory bowel disease, *IQR* interquartile range, *TL* trough level (expressed in μg/mL), *UC* ulcerative colitis

^a^Complete Mayo score of ≤2 points AND no individual subscore > 1 point (UC) OR CD activity score of ≤150 points

^b^Clinical response: reduction in complete or partial Mayo score of ≥3 points and ≥ 30% from baseline, AND a decrease of ≥1 point on the rectal bleeding subscore, OR an absolute rectal bleeding subscore ≤1; clinical remission: complete Mayo score of ≤2 points, AND no individual subscore > 1 point (UC)

^c^Absence of ulcers (CD) OR Mayo endoscopic subscore ≤1 (UC)

^d^Clinical effectiveness: physician global assessment; biological effectiveness: C-reactive protein level < 5 mg/L; endoscopic effectiveness: absence of ulcers (CD) OR Mayo endoscopic subscore ≤1 (UC)

^e^Absence of significant intestinal inflammation on magnetic resonance imaging, AND/OR absence of ulcers (CD), OR Mayo endoscopic subscore ≤1 (UC)

^f^Harvey Bradshaw Index < 5 (CD), OR partial Mayo score < 2 (UC), AND C-reactive protein level < 5 mg/L, AND no oral corticosteroid use in prior four weeks

^g^Thirty-five histological samples from 31 patients

^h^Nancy histological index ≤1

^i^Simple endoscopic score < 2 (CD), OR Mayo endoscopic subscore ≤1 (UC) while off steroids

### Clinical trials

Post-hoc analyses of the GEMINI trials were the first to reveal that higher vedolizumab trough levels during induction therapy correlated with higher clinical remission rates in IBD [9]. Interestingly, induction trough levels of less than 17 μg/mL for UC, and less than 16 μg/mL for CD, were associated with clinical remission rates similar to that of placebo. The exposure–efficacy relationship was steeper for UC than for CD [9]. The same authors recently published a propensity score-based case-matched analysis of UC patients in the GEMINI trials, aiming to characterise the relationship between vedolizumab exposure and response using patient-level data, and adjusting for confounding factors affecting vedolizumab drug clearance and serum levels [10]. Potential target vedolizumab trough levels were 37.1 μg/mL at week 6 during induction, 18.4 μg/mL at week 14 after induction, and 12.7 μg/mL during maintenance treatment [10].

### Real-world cohorts

Data from the two largest available real-world cohorts to date confirmed a link between higher vedolizumab exposure and achieving better outcomes [13, 15]. In a retrospective Belgian study, vedolizumab trough levels > 30 μg/mL at week 2, > 24 μg/mL at week 6, and > 14 μg/mL during maintenance therapy, correlated with higher clinical and endoscopic effectiveness endpoints in IBD patients [13]. Endoscopic remission was achieved in significantly more patients with UC than patients with CD, even though a diagnosis of UC was not an independent predictor of higher vedolizumab trough levels [13]. In a cross-sectional study from the USA, patients in steroid-free clinical and biologic remission had significantly higher vedolizumab trough levels than those who did not, although differences between both groups were small [15].

Multicentric data from France showed that vedolizumab trough levels below 18.5 μg/mL at week 6 were associated with the need for additional doses during the first 6 months of therapy [11]. A similar cut-off proved to be the only independent variable associated with mucosal healing within the first year of vedolizumab treatment [14]. In the latter study, only median vedolizumab trough levels at week 6, and not at week 2 or week 14, differed between patients with and without mucosal healing within the first year after treatment initiation [14]. A similarly designed study from another group also noted higher vedolizumab trough levels during induction in patients with steroid-free endoscopic remission after one year of treatment; however, only trough levels at week 2 differed significantly [17]. Interestingly, higher vedolizumab trough levels during maintenance therapy for UC have also been associated with histological remission, a distinct treatment target linked with better clinical outcomes [16]. Nevertheless, only a small number of patients were included, and confirmation in larger, independent cohorts remains necessary [16]. In conflict with the majority of data, one study found no exposure–efficacy relationship for vedolizumab [12].

## Pharmacokinetics/pharmacodynamics

Vedolizumab has a primarly linear clearance at therapeutic concentrations, with no difference between patients with CD and UC [18, 19]. Pooled population data from the GEMINI program identified low albumin concentration and very high body mass to be predictors of accelerated vedolizumab clearance [18]. Indeed, most real-world studies seem to confirm the clinical importance of these factors, linking them with lower drug levels and worse therapeutic outcomes [12, 13]. Low albumin also influences infliximab and adalimumab clearance rates, and is in part a surrogate marker of disease severity [20]. In this regard, more severe disease at initiation of vedolizumab therapy, reflected in low baseline albumin, but also in low haemoglobin and/or high C-reactive protein (CRP) levels, has been associated with lower vedolizumab drug levels during treatment and a lower likelihood of achieving therapeutic targets [9, 13, 17, 21]. There is an inverse relationship between vedolizumab drug levels and CRP [12, 14, 21, 22]. However, whether lower drug levels exacerbate disease activity, or whether higher disease activity results in lower drug levels, remains uncertain.

## Immunogenicity

Immunogenicity of vedolizumab in randomised controlled trials was low, with less than 5% of patients having at least one sample testing positive for anti-vedolizumab antibodies at any time, and fewer than 1% of patients with persistently positive anti-vedolizumab antibodies [4, 5]. This has been confirmed in all available real-world data so far, even when using a drug-resistant assay [23, 24]. It might explain why adding an immunomodulator to vedolizumab therapy seems neither to enhance drug levels nor to restore therapeutic response [14, 25].

## Promising features and potential drawbacks

**Fig. 1:**
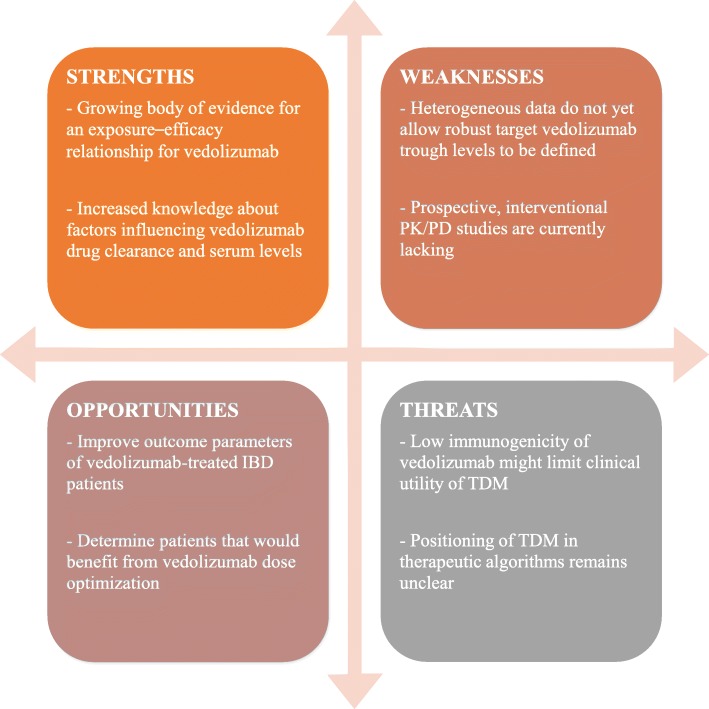
SWOT analysis (strengths, weaknesses, opportunities and threats) summarizing the promising features and potential drawbacks of vedolizumab trough level monitoring in inflammatory bowel disease (IBD). PK/PD: pharmacokinetic/pharmacodynamic; TDM: therapeutic drug monitoring

Despite the growing body of evidence for an exposure–efficacy relationship for vedolizumab, data remain difficult to interpret and do not allow firm recommendations to be drawn for clinical practice (Fig. 1). Most real-world studies were not designed prospectively with the primary aim of making inferences about the drug exposure–response profile. Heterogeneous study designs, including different outcome parameters and definitions, make direct comparisons difficult. Further more, all but one [10] study analysed data on a population level instead of an individual level, refraining to adjust for confounding factors that influence vedolizumab drug clearance and serum levels. Lastly, in the absence of comparative data between different assays used to measure vedolizumab drug levels, it cannot be ruled out that absolute differences in cut-offs across studies are secondary to disagreement of the utilised assays.

Therapeutic drug monitoring of TNF antagonists has shown to improve response and remission rates in IBD patients [26, 27]. It proved to aid mostly in determining the therapeutic strategy in anti-TNF-treated patients who are losing response [28], and led to major cost savings [29]. Extrapolation of these data to vedolizumab-treated patients is not yet possible because of the lack of robust evidence. Furthermore, the utility of therapeutic drug monitoring for anti-TNF has been strengthened by the likelihood of antidrug antibody development, since this should prompt clinicians to initiate a different TNF antagonist rather than to switch to another class of biological. Currently, no other anti-integrin is widely available, and antibody development against vedolizumab is a rare event, so the benefit of therapeutic drug monitoring for vedolizumab may be more marginal, and its positioning in therapeutic algorithms is yet to be defined.

## Future directions

The mechanisms underlying non-response or loss of response to vedolizumab are unrevealed. Almost complete occupancy of integrin α4β7 is seen on peripheral and intestinal T-cells from vedolizumab-treated IBD patients, regardless of serum levels [22]. Together with low immunogenicity, vedolizumab probably has additional modes of action. In this respect, vedolizumab treatment was recently shown to exert substantial effects on macrophage populations and expression of molecules involved in microbial sensing, chemoattraction and regulation of the innate effector response, suggesting at least a role for the innate immune system in the therapeutic efficacy of vedolizumab [30].

Dose optimisation of vedolizumab restores responsiveness in half of patients experiencing a secondary loss of response [31]. In a small, retrospective analysis of IBD patients who underwent vedolizumab dose optimisation, mean change from the baseline of vedolizumab trough levels at month 3 after dose optimisation was numerically higher in the group of responders versus the group of non-responders [32]. Before reactive therapeutic drug monitoring in vedolizumab-treated patients with insufficient response can be widely recommended, prospective studies must explore the effect of dose optimisation on objective disease markers and changes in vedolizumab drug levels. In this regard, the results of a continuing vedolizumab dose optimisation randomized controlled trial, ENTERPRET (NCT03029143), are eagarly awaited. This type of study should also allow further clarification of desired trough level intervals, both during induction and maintenance treatment.

Conflicting data mean the proactive use of therapeutic drug monitoring in symptom-free patients is heavily debated for anti-TNF [27, 33]. It remains to be seen whether cost-effectiveness for vedolizumab will ever be shown.

## Conclusion

Data from randomised registration trials and subsequent real-world cohorts suggest an exposure–efficacy relationship of vedolizumab in patients with CD and UC, but important drawbacks for therapeutic drug monitoring of vedolizumab still exist. Before therapeutic drug monitoring for vedolizumab can be widely recommended, prospective studies must evaluate the effect of vedolizumab dose optimization. These studies should focus on objective disease markers and vedolizumab drug levels, and define thresholds for optimal drug exposure.
